# Combined prediction of miR‐210 and miR‐374a for severity and prognosis of hypoxic–ischemic encephalopathy

**DOI:** 10.1002/brb3.835

**Published:** 2017-12-30

**Authors:** Zhansheng Wang, Yulu Liu, Minkun Shao, Dong Wang, Ying Zhang

**Affiliations:** ^1^ Department of Neonatal Intensive Care Unit The First People's Hospital of Shangqiu Shangqiu Henan China

**Keywords:** combined diagnosis, hypoxic–ischemic encephalopathy, miR‐210, miR‐374a, prognosis, receiver operating characteristic curve

## Abstract

**Background and Aim:**

Hypoxic–ischemic encephalopathy (HIE) is a disorder featured by hypoxic and ischemic damages during the perinatal period and its high mortality (i.e., 15%–20%) could be partly attributed to late diagnosis. Therefore, miR‐210 and miR‐374a were investigated to find if they could improve the diagnostic values of S100B protein and neuron‐specific enolase (NSE) for HIE.

**Methods:**

Altogether 167 HIE newborns and 82 healthy newborns were recruited, and their blood were sampled for determining the levels of biomarkers. Specifically, S100B protein and NSE levels were detected based on the enzyme‐linked immunosorbent assay (ELISA) kit, while the expressions of miR‐210 and miR‐374a were quantified by quantitative reverse transcription–polymerase chain reaction (qRT‐PCR). Moreover, the receiver operating characteristic (ROC) curves were established to assess the diagnostic values of the above biomarkers for HIE. Finally, the correlation analysis between miR‐210/miR‐374 and Neonatal Behavioral Neurological Assessment (NBNA) scoring or Gesell intellectual development were also conducted.

**Results:**

The levels of miR‐210, miR‐374a, S100B protein, and NSE were significantly distinct between HIE patients and healthy newborns (*p *<* *.05). Besides, miR‐210 (*r*_s_ = .573), miR‐374a (*r*_s_ = .651), NSE level (*r*_s_ = −.622), and S100B level (*r*_s_ = −.55) were all, respectively, correlated with NBNA scoring with statistical significance (*p *<* *.05). Furthermore, it was revealed that the combined diagnosis of miR‐210, miR‐374a, S100B protein, and NSE could obtain the highest accuracy regarding pairs of mild HIE versus moderate HIE (AUC = 0.898), moderate HIE versus severe HIE (AUC = 0.922), mild HIE versus severe HIE (AUC = 0.996), and HIE versus control (AUC = 0.960). More than that, the four molecules were also remarkably associated with Gesell intellectual development (*p *<* *.05).

**Conclusion:**

MiR‐210 and miR‐374a could help to elevate the diagnostic value and prognostic prediction of S100B protein and NSE for HIE.

## 1 INTRODUCTION

Hypoxic–ischemic encephalopathy (HIE) is generally defined as the hypoxic and ischemic damages caused by anoxia asphyxia during the perinatal period, and its pathological manifestations are mainly the changes of cerebral blood flow (CBF) and brain tissue metabolism (Fukuda et al., 2008; Liu, Liu, Lu, Liu, & Jiang, 2015). The Chinese incidence of HIE varies between 3‰ and 6‰ among 18–20 million live births per year, and around 15%–20% of the HIE infants ultimately die in the newborn period (Shao, Ye, & Qiu, 2011). According to the multicentric studies from domestic and overseas groups, mild hypothermia remains the therapeutic measure that could gain a relatively favorable clinical efficacy for HIE (Zhou et al., 2010). Nevertheless, with this treatment, infants with minor and moderate HIEs could return to normal within 10 days, while those with severe HIE lesions are observed with echo‐free space (i.e., cystic degeneration) in 2–3 months and high possibility of death. Therefore, early diagnosis of HIE are critical to lessen the neurological sequel and to decrease the neonatal mortality (Graham, Ruis, Hartman, Northington, & Fox, 2008). Currently, HIE is diagnosed partly depending on observing the infants’ clinical symptoms, yet this process takes a long time and is vulnerable to subjectivity. Application of computed tomography (CT) and magnetic resonance imaging (MRI) for diagnosing HIE is limited by peoples’ fear of radioactive rays and the high inspection charges (Jose, Matthai, & Paul, 2013; Zhang, Zhang, & Li, 2016). In response, it is crucial to discover the sensitive and specific biomarkers that could objectively diagnose the early‐stage HIE in a cost‐effective manner.

Multiple biomarkers that could identify the newborns’ central nervous system lesions have been investigated, for instance, S100B protein, a calcium‐binding protein that was principally distributed within certain neurons, melanophores and adipocytes, were closely linked with severe cerebral injury, and the increased synthesis of S100B could repair the nerve injury (Goncalves, Leite, & Guerra, 2010) (Goncalves, Leite, & Nardin, 2008; Sun, Liu, & Nie, 2013). In addition, S100B proteins would be released into cerebral spinal fluid (CSF) and serum when necrosis of neurogliocytes and damage of brain–blood barrier (BBB) happened, suggesting that high S100B protein levels might be representative of poor HIE prognosis (Okumus et al., 2008). Similar to S100B protein, neuron‐specific enolase (NSE) that was supposed to be within brain tissues would be discharged into CSF after HIE development, implying its significance in symbolizing the onset of HIE (Douglas‐Escobar & Weiss, 2012; Shimono et al., 2010). Nevertheless, the sensitivity and specificity of S100B and NSE were still finite, when they were, respectively, utilized for HIE diagnosis (Azuma et al., 2015; Gazzolo et al., 2009).

Interestingly, it was revealed that miR‐374a expressions were remarkably reduced within HIE infants in comparison to healthy infants and ones with perinatal asphyxia (Looney et al., 2015). Furthermore, miR‐210 was demonstrated to exert neuroprotective effects on HIE murine via modifying the bcl‐2/bax balance and thereby the cell apoptosis (Qiu et al., 2013). Since microRNAs have been deemed as the desirable biomarkers for diverse disorders (e.g., cancer) (Morimura et al., 2011; Su et al., 2014; Wang et al., 2014), it was hypothesized that miR‐374a and miR‐210 might also serve as potential diagnostic biomarkers for HIE patients.

On the whole, since diagnosing of HIE with single biomarkers could contribute to relatively low sensitivity and specificity, the present study was aimed to explore whether the synergic utilization of miR‐374a and miR‐210 could improve the diagnostic value of S100B protein and NSE for HIE patients.

## 2 METHODS

### 2.1 Subjects

From January 2015 to December 2016, totally 167 HIE newborns and 82 healthy newborns were recruited from the First People's Hospital of Shangqiu, and the newborns were all guaranteed to be within 48 hr after their births. The full‐term infants were diagnosed as HIE in accordance with Sarnat and Sarnat (1976), whereas the premature infants were diagnosed if they have medical history of anoxia asphyxia and typical neurological symptoms, including overexcitation, drowsiness, coma, dystonia, abnormality in primitive reflex, convulsions, and so on (Chen, 2004; Chen & Yu, 2004). Of note, the infants mentioned above were confirmed with HIE after conduction of brain magnetic resonance imaging (MRI) (Cowan et al., 2003). The above HIE subjects were classified into mild, moderate, and severe grades according to the diagnostic criteria of neonatal HIE (Table S1) (Group of Neonatology CPS, & Chinese Medical Association, 2005). Moreover, the healthy newborns should not be accompanied by medical history of perinatal asphyxia or abnormal nervous system after birth. For both HIE infants and healthy newborns, the subjects would be removed if they suffered from convulsions caused by electrolyte disturbance, intracranial hemorrhage, and birth injury. Besides, the participants with brain injury caused by intrauterine infection, hyperbilirubinemia, and severe brain dysplasia were also excluded (Krsek et al., 2010; Stevenson, Benitz, Sunshine, Hintz, & Druzin, 2009; Zhao, Chen, Xu, & Pi, 2013). Ultimately, all the infants’ parents have signed the informed consents, and this study was approved by the institution review board (IRB) and the ethics committee of the First People's Hospital of Shangqiu (Henan province).

### 2.2 Detection of NSE and S100B protein

Since the damages of neuronal cells and the metabolism of energy substrates were the most pronounced within 48 hr after the occurrence of anoxia (Whitelaw & Thoresen, 2010), NSE and S100B were collected from the newborns within 6 hr after their births. Blood sampling was conducted via femoral vein in the morning, and all subjects should be on their empty stomachs. The coagulated blood (volume: 2 ml) were examined within 48 hr since delivery. The levels of serum NSE and S100B protein were detected with ELISA kit (CanAg Corporation, Sweden), and were managed with type 550 microplate reader (Bio‐Rad Corporation, USA).

### 2.3 Detection of miR‐210 and miR‐374a with real‐time PCR

Umbilical cord blood (volume: 3 ml) was gathered within 1 hr since delivery. After the umbilical cord blood was centrifuged at the speed of 3,500 rpm for 10 min, its serum was kept in the −80°C refrigerator. Total RNAs were isolated in line with the specifications of miRNeasy‐Mini‐Kit (Qiagen), and they were examined within 48 hr since delivery. The first cDNA strands were synthesized based on M‐MLV reverse transcriptase, and quantitative PCR reaction was carried out based on the miRNA RT‐PCR Kit (Takara) and ABI7300 system. With the primers of miR‐374a, miR‐210, and U6 (Guangzhou Ruibo Biotechnological Corporation, China), the PCR reaction was advanced in accordance with the following procedures: (i) predegeneration at 95°C for 30 s and (ii) 40 cycles of 95°C for 5 s, 55°C for 30 s, and 72°C for 30 s. Then, ABI7300 real‐time PCR software was arranged to analyze the relative copy number of genes. During the course of the experiment, the contamination of proteins within RNAs would be considered as tolerable when the ratios of OD260 and OD280 ranged between 1.8 and 2.1. Besides, the complexity of RNAs was evaluated according to the RNA Integrity Number (RIN) of the software, and the capillary electrophoresis was conducted with Agilent Bioanalyzer.

### 2.4 Treatment

The head local cooling was mainly applied to treat the HIE newborns via the model RC‐2000 hypothermia instrument (RiCheng Medical Electronic Equipment Limited Corporation, Jilin province, China). Specifically, therapeutic caps were put on the head of patients, with recycled water prepared for cooling the head. This treatment was started in the sixth hour after birth, and the nasopharyngeal temperature was maintained at 34 ± 0.2°C for 72 hr under the computer automatic control. After the cease of head cooling, natural rewarming was implemented, and infrared radiation was applied for ones whose body temperature was below 36°C after 6 hr.

### 2.5 NBNA scoring

All the detection procedures were processed in the quiet and half‐dark room at the temperature of 22–27°C. The behavioral neurology of full‐term infants was assessed by trained physicians on the seventh day after their births, while the Neonatal Behavioral Neurological Assessment (NBNA) scoring of premature infants was evaluated at term (Li et al., 2008; Zhu et al., 2015). The scoring was implemented 1 hr after breastfeeding, and the total inspection time was guaranteed to be within 10 min. The evaluation items of NBNA could be generally divided into five parts, including active ability (*n* = 6), passive muscle tension (*n* = 4), active muscle tension (*n* = 4), primitive reflex (*n* = 3), and general evaluation (*n* = 3). It would be graded as: (i) low when the score was set as 0, (ii) medium when the score was set as 1, and (iii) high when the score was set as 2. Besides, the newborns would be considered as: (i) the higher NBNA group when their NBNA score was above 37, (ii) the lower NBNA group when their NBNA score was <35, and (iii) the medium NBNA group when their NBNA score ranged between 35 and 37.

### 2.6 Evaluation of Gesell intellectual development

HIE newborns all underwent intellectual measurements, respectively, in the 48th and 52nd weeks after their births. The evaluation criteria were in light of the Gesell intellectual development scale, and the development quotients (DQs) of five energy regions were checked, including adaptive behavior, large motion, fine movement, language, and personal social behavior (Zhu & Zhu, 1985).

### 2.7 Statistical analysis

All the statistical analyses were completed throughout SPSS 17.0 software. The numerical variables indicated in the form of mean ± standard deviation (*SD*) were compared utilizing analysis of variance (ANOVA), when they accorded with normal distribution. The categorical variables presented in the form of proportion were analyzed with chi‐square test. Moreover, the correlative relationship between parameters was figured out with application of linear correlation and regression analysis. Finally, receiver operating characteristic (ROC) curve was fitted to calculate the area under ROC curve (AUC), and the diagnostic sensitivity and specificity of biomarkers were identified based on Youden index. It would be considered as statistical significance when *p* value was <.05.

## 3 RESULTS

### 3.1 Baseline characteristics of included subjects

The mild‐HIE newborns and normal newborns were observed to share similar sex ratio, eutocia/cesarean ratio, mean weight, and mean gestational age (all *p*s* *>* *.05; Table 1). The differences between medium and severe HIE newborns regarding male/female ratio, eutocia/cesarean ratio, mean weight, and mean gestational age were also statistically insignificant (all *p*s* *>* *.05).

**Table 1:** Baseline characteristics of included subjects

Characteristics	Healthy	Mild HIE	Moderate HIE	Severe HIE
Male	45	34	33	18
Female	40	35	27	20
Chi‐square test	Ref	0.205	0.060	0.326
*p* value	Ref	.651	.807	.598
Eutocia	47	38	32	23
Cesarean	38	31	28	15
Chi‐square test	Ref	0.001	0.055	0.293
*p* value	Ref	.978	.815	.588
Weight	3,691 ± 413	3,728 ± 467	3,609 ± 540	3,785 ± 462
*t* test	Ref	.521	1.036	1.124
*p* value	Ref	.603	.302	.263
Gestational age	39.14 ± 0.26	38.98 ± 1.05	39.07 ± 0.34	38.97 ± 0.79
*t* test	Ref	1.356	1.404	1.787
*p* value	Ref	.177	.162	.077

HIE, Hypoxic–ischemic encephalopathy; Ref, reference.

### 3.2 Comparison of NSE, S100B, miR‐210, and miR‐374a levels between HIE newborns and healthy controls

The healthy newborns possessed significantly less NSE and S100B levels, yet higher miR‐210 and miR‐374a levels than HIE newborns (all *p*s* *<* *.05; Table 2). HIE newborns were also associated with significantly higher NBNA scores than healthy newborns (*p *<* *.05). Moreover, with incremental severity of HIE, NSE and S100B levels were remarkably increased, whereas miR‐210 and miR‐374a levels were significantly downregulated (*p *<* *.05). The NBNA scores were also decreased when HIE turned severe (*p *<* *.05).

**Table 2:** Comparison of NSE, S100B, miR‐210, and miR‐374a levels between HIE and healthy newborns

Groups	Grading	*N*	NSE (μg/L)	S100B (μg/L)	miR‐210	miR‐374a	NBNA
HIE group	Mild	69	38.09 ± 22.82	0.82 ± 0.51	1.02 ± 0.21	0.99 ± 0.32	36.13 ± 1.57
	Moderate	60	53.62 ± 24.83	1.20 ± 0.59	0.84 ± 0.16	0.64 ± 0.26	33.42 ± 1.61
	Severe	38	74.77 ± 29.19	1.98 ± 0.64	0.67 ± 0.19	0.33 ± 0.29	31.74 ± 1.25
Healthy group	—	85	15.53 ± 6.41	0.54 ± 0.32	1.15 ± 0.14	1.26 ± 0.33	38.56 ± 1.82

HIE, Hypoxic–ischemic encephalopathy; NSE, neuron‐specific enolase; NBNA, Neonatal Behavioral Neurological Assessment.

Furthermore, the NSE and S100B levels in the higher NBNA group were significantly higher than those in the medium NBNA group, and the medium group embraced higher NSE and S100B levels than the low NBNA group (*p *<* *.05; Table 3). On the contrary, miR‐210 and miR‐374a levels in the higher NBNA group were significantly reduced in comparison to those in the medium NBNA group, and the medium NBNA group was accompanied with lower NSE and S100B levels when compared with the lower NBNA group (*p *<* *.05).

**Table 3:** Comparison of NSE, S100B, miR‐210, and miR‐374a levels between HIE newborns grouped on NBNA score

NBNA score	*N*	NSE (μg/L)	S100B (μg/L)	miR‐210	miR‐374a
≥37	87	12.29 ± 5.86	0.52 ± 0.36	1.25 ± 0.22	1.23 ± 0.24
37–35	75	47.11 ± 17.36	0.92 ± 0.53	0.93 ± 0.17	0.89 ± 0.21
≤35	90	60.05 ± 21.42	1.51 ± 0.57	0.73 ± 0.16	0.58 ± 0.19

HIE, Hypoxic–ischemic encephalopathy; NSE, neuron‐specific enolase; NBNA, Neonatal Behavioral Neurological Assessment.

Besides, either NSE level (*r*_s_ = −.622) or S100B level (*r*_s_ = −.550) was negatively correlated with NBNA scoring (*p *<* *.05), whereas miR‐210 (*r*_s_ = .573) and miR‐374a (*r*_s_ = .651) were both positively associated with NBNA scoring (*p *<* *.05; Fig. S1).

### 3.3 Diagnostic performance of NSE, S100B, miR‐210, and miR‐374a levels for HIE newborns

MiR‐374a topped with respect to differentiating moderate (AUC = 0.806) or severe HIE (AUC = 0.930) from mild HIE (Table 4). In addition, the NSE level was preferred in differentiating HIE patients from healthy controls (AUC = 0.899), and S100B protein level was mostly recommended to differentiate moderate HIE from severe HIE (AUC = 0.878).

**Table 4:** Diagnostic performance of NSE, S100B, miR‐210, and miR‐374a levels for HIE newborns

Biomarkers	Comparison	Value	SEN	SPE	AUC	95% CI
NSE	Mild vs. Moderate	>61.90	40.0%	87.0%	0.67	0.58–0.77
	Moderate vs. Severe	>65.20	73.7%	65.0%	0.72	0.61–0.83
	Mild vs. Severe	>63.37	73.7%	87.0%	0.83	0.74–0.92
	HIE vs. Control	>26.15	78.4%	94.1%	0.90	0.86–0.94
S100B	Mild vs. Moderate	>1.05	60.0%	30.4%	0.68	0.59–0.77
	Moderate vs. Severe	>1.64	71.1%	83.3%	0.88	0.73–0.90
	Mild vs. Severe	>1.53	78.9%	91.3%	0.92	0.87–0.98
	HIE vs. Control	>0.80	69.5%	81.2%	0.80	0.74–0.85
miR‐210	Mild vs. Moderate	<0.97	81.7%	62.3%	0.76	0.67–0.84
	Moderate vs. Severe	<0.56	42.1%	98.3%	0.73	0.63–0.84
	Mild vs. Severe	<0.84	78.9%	85.5%	0.90	0.84–0.95
	HIE vs. Control	<1.01	73.1%	84.7%	0.84	0.80–0.89
miR‐374a	Mild vs. Moderate	<0.95	90.0%	63.8%	0.81	0.73–0.88
	Moderate vs. Severe	<0.53	78.9%	68.3%	0.79	0.69–0.88
	Mild vs. Severe	<0.61	86.8%	88.4%	0.93	0.88–0.98
	HIE vs. Control	<0.90	67.7%	87.1%	0.86	0.81–0.90

HIE, Hypoxic–ischemic encephalopathy; NSE, neuron‐specific enolase; SEN, sensitivity; SPE, specificity; AUC, area under the curve; CI, confidence interval.

**Figure 1:**
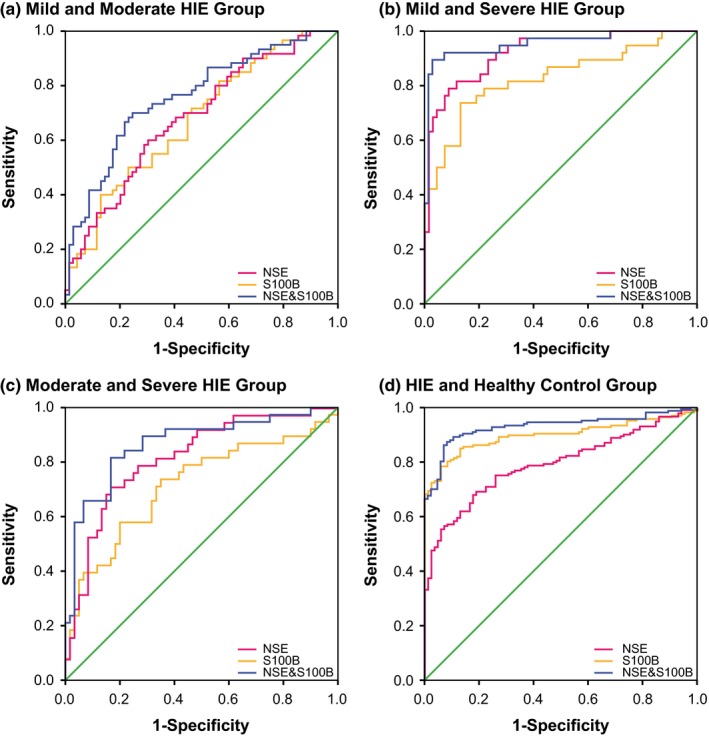
Combination diagnostic performance of NSE and S100B protein for HIE newborns

**Figure 2:**
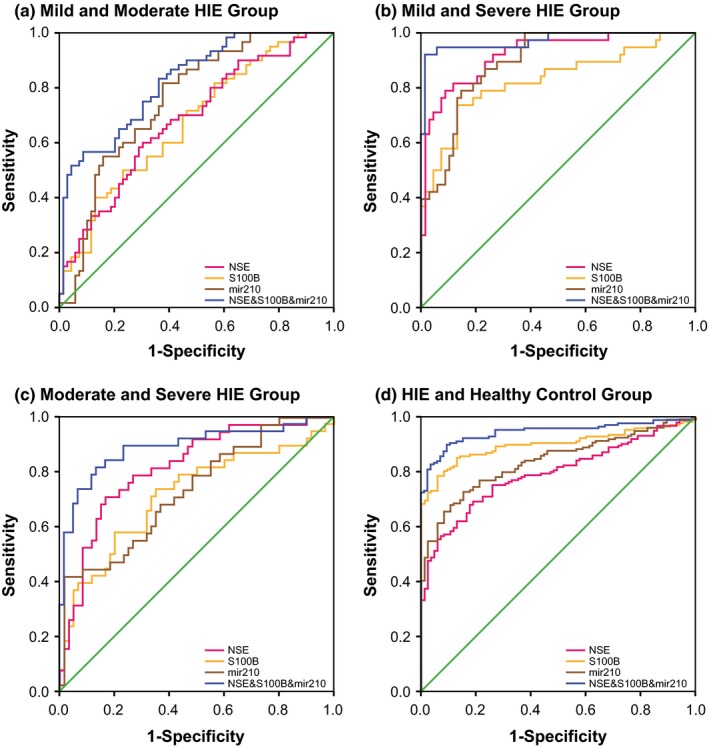
Combination diagnostic performance of NSE, S100B protein, and miR‐210 level for HIE newborns

**Figure 3:**
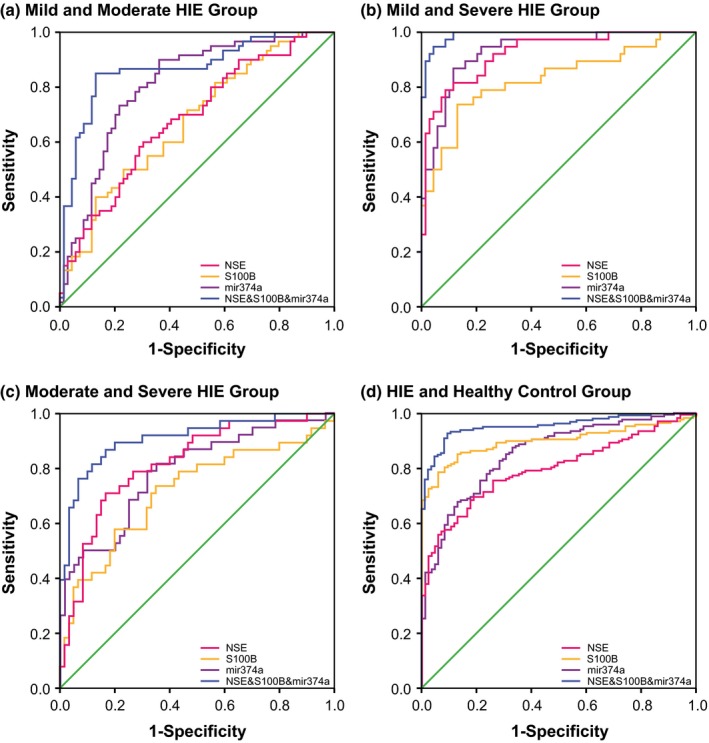
Combination diagnostic performance of NSE, S100B protein, and miR‐374a level for HIE newborns

**Figure 4:**
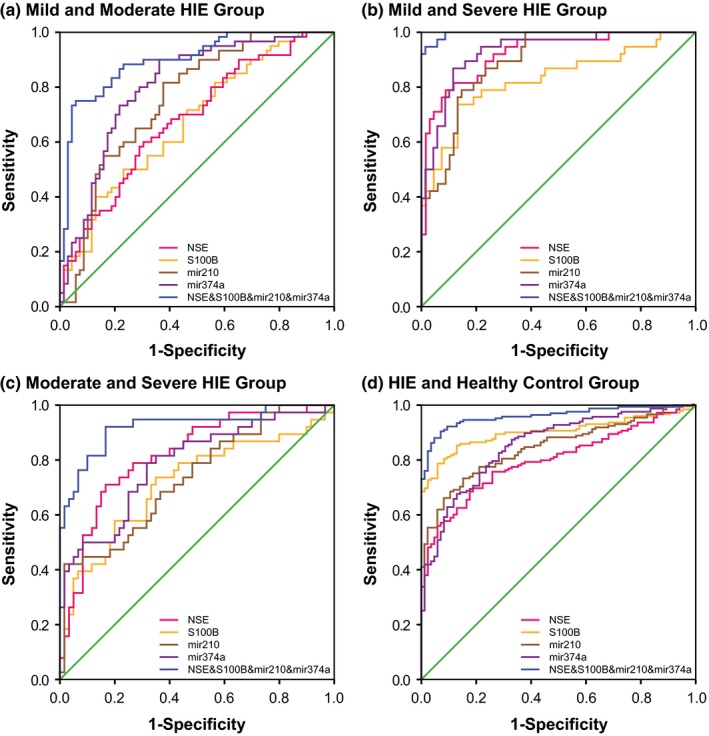
Combination diagnostic performance of NSE, S100B protein, miR‐210, and miR‐374a levels for HIE newborns

As for the combined diagnosis (Figures 1, 2, 3, 4), the synthetic role of NSE, S100B, miR‐210, and miR‐374a peaked when comparisons of mild HIE versus moderate HIE (AUC = 0.898), moderate HIE versus severe HIE (AUC = 0.922), mild HIE versus severe HIE (AUC = 0.996), and HIE versus control (AUC = 0.960) were considered (Table 5). Then, the NSE + S100B + miR‐374a, NSE + S100B + miR‐210, and NSE + S100B groups were, respectively, ranked No. 2, No. 3, and No. 4 in their diagnostic performance concerning the above four comparisons.

**Table 5:** Combination diagnostic performance of NSE, S100B, miR‐210, and miR‐374a levels for HIE newborns

Combination biomarkers	Comparison	SEN	SPE	AUC	95% CI
NSE and S100B	Mild vs. Moderate	70.00%	75.40%	0.76	0.68–0.84
	Moderate vs. Severe	81.60%	83.30%	0.87	0.79–0.94
	Mild vs. Severe	89.50%	97.10%	0.96	0.91–1.00
	HIE vs. Control	87.40%	91.80%	0.93	0.90–0.96
NSE + S100B + miR‐210	Mild vs. Moderate	56.70%	91.30%	0.82	0.75–0.89
	Moderate vs. Severe	81.60%	86.70%	0.89	0.82–0.96
	Mild vs. Severe	92.10%	98.60%	0.97	0.94–1.00
	HIE vs. Control	89.80%	90.60%	0.94	0.92–0.97
NSE + S100B + miR‐374a	Mild vs. Moderate	85.00%	87.00%	0.87	0.80–0.93
	Moderate vs. Severe	86.80%	83.30%	0.91	0.85–0.97
	Mild vs. Severe	94.70%	95.70%	0.99	0.98–1.00
	HIE vs. Control	92.80%	90.60%	0.96	0.93–0.98
NSE + S100B + miR‐210 + miR‐374a	Mild vs. Moderate	75.00%	94.20%	0.90	0.84–0.95
	Moderate vs. Severe	92.10%	83.30%	0.92	0.86–0.98
	Mild vs. Severe	94.70%	98.60%	1.00	0.99–1.00
	HIE vs. Control	90.40%	92.90%	0.96	0.94–0.98

HIE, Hypoxic–ischemic encephalopathy; NSE, neuron‐specific enolase; SEN, sensitivity; SPE, specificity; AUC, area under the curve; CI, confidence interval.

### 3.4 Comparison of Gesell intellectual development between HIE and healthy newborns

The normal newborns always overweighed HIE ones in both 48‐week and 52‐week DQs (*p *<* *.05), and the DQ values decreased significantly with severity of HIE (*p *<* *.05; Table 6). Moreover, NSE and S100B levels were negatively correlated with 48‐week and 52‐week DQs (*p *<* *.05), whereas miR‐210 and miR‐374a were positively correlated with 48‐week and 52‐week DQs (*p *<* *.05; Figs [Link], [Link]). Furthermore, it was indicated that the predictive role of combined biomarkers (i.e., miR‐210, miR‐374a, NSE, and S100B) for 48‐week and 52‐week DQs was more accurate than that of any single biomarker, and the AUC values of miR‐210 and miR‐374a were higher than those of NSE and S100B.

**Table 6:** Comparison of Gesell intellectual development between HIE and healthy newborns

Groups	Grading	*N*	48 weeks DQ scores		52 weeks DQ scores	
			Mean	*SD*	Mean	*SD*
HIE group	Mild	69	85.31	8.74	87.85	6.12
	Moderate	60	79.61	6.07	83.22	5.34
	Severe	38	74.58	7.26	76.14	7.51
Healthy group	—	85	88.29	5.47	89.66	9.09

HIE, Hypoxic–ischemic encephalopathy; DQ, development quotient.

## 4 DISCUSSION

In the case of HIE, since CSF‐NSE was increased when neurons turned degenerative and necrotic, and the serum NSE (s‐NSE) level was elevated due to BBB disruption, both s‐NSE and CSF‐NSE could be deemed as the biomarkers for indicating the severity and prognosis of neuron damages (Noaman et al., 2013; Varsami et al., 2013). For instance, when the cut‐off value of NSE was set as ≥45.4 μg/L, the sensitivity and specificity of s‐NSE for diagnosing medium and severe HIE were, respectively, 79% and 70% (Streitberger et al., 2017). However, applying NSE for diagnosing HIE was still unsatisfactory, for that the increased NSE could also be found within other disorders, including neonatal hyperbilirubinemia, infectious brain injury, and brain injury caused by congenital metabolic diseases (Gazzolo et al., 2009; Yao, Zhang, Ai, Liu, & Huang, 2014).

Regarding S100B protein, it was usually detected within blood, CBF, saliva, amniotic fluid, and breast milk, and its increase could precisely predict severity and prognosis of brain injury (Morochovic et al., 2009). In particular, Okumus et al. (2008) discovered that S100B level might have something to do with the neurological system lesions, which have been ascertained by electroencephalogram (EEG) and brainstem auditory evoked potential (BAEP). Moreover, patients with S100B level ≥4 μg/L were usually accompanied by serious hemorrhagic brain injury, yet ones with S100B level <4 μg/L were prone to suffer from diffusive embolic cerebral infarction (Korfias et al., 2007). Intriguingly, S100B protein was also proposed as the peripheral marker of BBB, owing to that the S100B level not only followed the time evolution rule within peripheral blood after the presence of BBB, but also was closely linked with the opening level of BBB (Koh & Lee, 2014; Weiss et al., 2015). All in all, the above phenomena and mechanisms might well explain why S100B level could reflect the HIE development and prognosis, as demonstrated in our investigation.

Furthermore, miR‐210 was documented to hinder neuronal apoptosis via downregulating the caspase activity and altering the bcl‐2/bax balance, although the protective role of miR‐210 was diminished when neonatal hypoxic–ischemic injury occurred (Qiu et al., 2013). Delivering miR‐210 into the ischemic heart also facilitated angiogenesis and suppressed apoptosis, ultimately improving the heart function (Hu et al., 2010). Furthermore, the expressions of miR‐210 and miR‐374a were found to differ significantly between HIE infants and healthy controls among an Irish population (Looney et al., 2015). Nonetheless, as people of discrepant ethnicities might embrace different genes and produce distinct biochemical effects, the study result might not be appropriate for the Chinese cohorts. The downstream targets of miR‐374a, such as activin‐A and activin‐A receptor type IIb (ACVR2B), were also documented to be potential biomarkers for predicting the severity of HIE among the Irishmen (Looney, Ahearne, Hallberg, Boylan, & Murray, 2016). To sum up, considering the roles of NSE, S100B, miR‐210, and miR‐374a in regulating the HIE mechanism, this study assessed their diagnostic performance for HIE among a Chinese population, drawing a conclusion that the synthetic role of NSE, S100B, miR‐210, and miR‐374a could reach a most ideal result regarding HIE diagnosis and prediction of HIE prognosis (i.e., DQ score).

Although the current study has enriched the diagnostic approaches for HIE, it was constrained in not categorizing the brain injury. That was to say, maybe specific assortments of molecules among S100B protein, NSE, miR‐210, and miR‐374a could achieve a higher accuracy for particularized brain injury. Besides, this study might be criticized for its utilizing four biomarkers, making the diagnostic procedures complicated and tedious. However, in future, the detection techniques tended to be fully automated, and people's health would be supreme, which would overshadow the seeming disadvantage. In addition, the sample size of this study was finite, and this study merely investigated the Chinese ethnicity, which made it tough to generalize this study result to a wide range of population. More than the above, as ubiquitin C‐terminal hydrolase‐L1 (UCH‐L1) and glial fibrillary acidic protein (GFAP) have been widely recognized as the characteristic biomarker for HIE neonates, their related combined diagnoses should also be followed closely (Douglas‐Escobar et al., 2014; Jiang, Wang, Zhang, & Jiang, 2014). Finally, in the subsequent researches, long‐term prognosis (e.g., 72‐week DQ score) for the HIE patients should also be observed, and the confounding effects of prematurity and multiple illnesses should also be considered. In consequence, to further validate the study result, a large‐scale study covering diverse ethnicities and targeting specific brain injuries should be conducted.

## CONFLICT OF INTEREST

None declared.
