# Supplementary material for: Combined prediction of miR‐210 and miR‐374a for severity and prognosis of hypoxic–ischemic encephalopathy
Source: Brain Behav. 2017 Dec 30;8(1):e00835. doi: 10.1002/brb3.835 (PMC5853646; doi:10.1002/brb3.835)
Supplement: Supplementary file 4 [file BRB3-8-e00835-s004.docx]

**Supplementary table 1 The clinical grading for Hypoxic ischemic encephalopathy.**

| **Grading** | **Mild** | **Moderate** | **Severe** |  |
| --- | --- | --- | --- | --- |
| **Consciousness** | Alteration of excitement and depression | Drowsiness | Coma |  |
| **Muscular tension** | Normal or a little higher | Lower | Soft or intermittent increased | |
| **Primitive reflex** |  |  |  |  |
| **Moro reflex** | Active | weak | diappeared | |
| **Sucking reflex** | Normal | weak | diappeared | |
| **Convulsion** | Myoclonus | Frequent | be in a continuous state | |
| **Central respiratory failure** | With | Without | Obvious |  |
| **Pupil change** | Normal or enlarged | Often narrowed | Asymmetric or enlarged, be obtuse to the light reflex | |
| **EEG** | Normal | Low pressure | Burst suppression | |
| **Course of disease** | Disappearance of symptoms within 72 h | Disappearance of symptoms within 14 d | Lasting of symptoms for several weeks | |
| **Prognosis** | Well | Could be with sequel | High mortality and high incidence of sequel for survivors | |
